# Making Theory Reasoning Simpler

**DOI:** 10.1007/978-3-030-72013-1_9

**Published:** 2021-02-26

**Authors:** Giles Reger, Johannes Schoisswohl, Andrei Voronkov

**Affiliations:** 8 Eindhoven University of Technology, Eindhoven, The Netherlands; 9 Aalborg University, Aalborg East, Denmark; 10 University of Manchester, Manchester, UK; 11 EasyChair, Manchester, UK

## Abstract

Reasoning with quantifiers and theories is at the core of many applications in program analysis and verification. Whilst the problem is undecidable in general and hard in practice, we have been making large pragmatic steps forward. Our previous work proposed an instantiation rule for theory reasoning that produced pragmatically useful instances. Whilst this led to an increase in performance, it had its limitations as the rule produces ground instances which (i) can be overly specific, thus not useful in proof search, and (ii) contribute to the already problematic search space explosion as many new instances are introduced. This paper begins by introducing that specifically addresses these two concerns as it produces general solutions and it is a simplification rule, i.e. it replaces an existing clause by a ‘simpler’ one. Encouraged by initial success with this new rule, we performed an experiment to identify further common cases where the complex structure of theory terms blocked existing methods. This resulted in four further simplification rules for theory reasoning. The resulting extensions are implemented in the Vampire theorem prover and evaluated on SMT-LIB, showing that the new extensions result in a considerable increase in the number of problems solved, including 90 problems unsolved by state-of-the-art SMT solvers.

## 1 Introduction

Many applications of reasoning in program analysis and verification depend on reasoning with the first-order theory of arithmetic, often in combination with other theories and quantifiers. A common approach to this problem is via Satisfiability Modulo Theory (SMT) solving, which has strong support for decidable theories but may struggle to scale in the presence of quantifiers. Conversely, superposition-based first-order solvers handle quantifiers naturally and have, recently, been extended to reason with theories [2,3,5,6,9,13,16,21]. Such solvers are based on a *saturation loop* and tend to suffer from *search space explosion.* This is compounded by the effective but explosive use of *theory axioms*, leading to the derivation of numerous inconsequential consequences of the theory. So far we have attempted to control this explosive behaviour [10,17] but now we aim to eliminate some of it. This paper introduces a set of *simplification rules* for reasoning in the theory of (any combination of linear or non-linear real, rational, or integer) arithmetic, i.e. rules that make reasoning in arithmetic simpler.

This work was motivated by our previous attempt [20] to find useful instances of first-order clauses that would be otherwise difficult to find via reasoning with theory axioms. For example, when considering the two clauses

$$r(7x) \qquad \neg r(6+y) \vee p(y)$$

our previous work would apply resolution on $r(7x)$ and $\neg r(6+y)$ using *unification with abstraction* to produce the clause $7x \neq 6+y \vee p(y)$ and then applied *theory instantiation*, utilising an SMT solver to find the substitution $\{x \mapsto 1, y \mapsto 1\}$, producing the instance $p(1)$. This may or may not be useful to proof search and, crucially, we need to keep performing inferences with the original clauses in case it is not. In this case, we would prefer to instantiate with $\{y \mapsto 7x-6\}$ to produce $7x \neq 6+(7x-6) \vee p(7x-6)$, which can be reduced to $p(7x-6)$. This is a *general* solution (being logically equivalent) that is also *simpler* – in this case it has fewer variables than the original clause. Hence, we replace the clause by the more general result, aiding proof search and preventing the addition of unnecessary instances.

The above was motivated by the observation that we would often see clauses of the form $\widehat{k}x \neq t \vee C[x]$ (for numeral $\widehat{k}$, variable $x$, and term $t$) and expend much effort using theory axioms to rewrite $\widehat{k}x \neq t$ into $x \neq \frac{t}{\widehat{k}}$. This led us to conduct an experiment to identify other common cases where arithmetic clauses could be simplified. An immediate observation is that, if $x$ ranges over the reals, $p(7x-6)$ can be instantiated with $\{x \mapsto \frac{(y+6)}{7}\}$ to produce $p(y)$. Furthermore, in the above example we no longer need to employ the expensive unification with abstraction as we can instantiate $r(7x)$ with $\{x \mapsto \frac{z}{7}\}$ to produce $r(z)$ and then resolve with $r(6+y) \vee p(y)$ to produce $p(y)$ directly.

Another observation was that a large amount of effort was expended by the theorem prover reordering sums and products to expose seemingly obvious structure. For example, taking $(3t+x)+2t$ and producing $5t+x$ requires three theory axioms and 12 rewriting steps. To combat this, we introduce an evaluation method that flattens sums and products, reorders and simplifies them, before reintroducing the necessary bracketed structure. A related common issue was the occurrence of terms that could easily be *cancelled*, such as in $4x+3 < 4x+10$, again requiring significant rewriting effort that can be replaced by a special rule.

This paper does not present the exploratory experimentation described above but focusses instead on the fruits of this work. After introducing the necessary preliminaries (Sec. 2), we make the following contributions:

– A new *Gaussian Variable Elimination* rule (Sec. 3) that eliminates variables if they can be described completely in terms of other variables.
– A set of *Arithmetic Subterm Generalisation* rules (Sec. 4) that replace clauses with obvious generalisations, as in the above cases of replacing $p(7x-6)$ with $p(y)$ and $r(7x)$ with $r(x)$.
– A general approach to the *evaluation* of terms involving arithmetic (Sec. 5), including a special rule to handle a surprisingly common corner case involving unary minus.

– A rule for *cancelling* subterms, e.g. in $4x + 3 < 4x + 10$ (Sec. 6)

These rules are all implemented in the Vampire [1,14] theorem prover. Our experimental evaluation (Sec. 7) shows that the new rules significantly improve the number of problems (from SMT-LIB) that Vampire can solve. Our final experiment shows that the new Vampire can solve 1,052 problems unsolved by Vampire 4.5, 1,056 problems unsolved by CVC4, and 1,350 problems unsolved by Z3 — given their complementary nature, this equates to 90 problems unsolved by any of these state-of-the-art solvers.

## 2 Preliminaries and Related Work

*First-Order Logic and Theories.* We consider a many-sorted first-order logic with equality. A *signature* is a pair $\Sigma = (\Xi, \Omega)$ where $\Xi$ is a set of *sorts* and $\Omega$ a set of *predicate* and *function* symbols with associated argument and return sorts from $\Xi$. *Terms* are of the form $c$, $x$, or $f(t_1, \ldots, t_n)$ where $f$ is a *function symbol* of arity $n \geq 1$, $t_1, \ldots, t_n$ are terms, $c$ is a zero arity function symbol (i.e. a constant) and $x$ is a variable. We assume that all terms are well-sorted and write $t : \sigma$ if term $t$ has sort $\sigma$. Atoms are of the form $p(t_1, \ldots, t_n), q$ or $t_1 \simeq_s t_2$ where $p$ is a predicate symbol of arity $n$, $t_1, \ldots, t_n$ are terms, $q$ is a zero arity predicate symbol and for each sort $s \in \Xi$, $\simeq_s$ is the *equality symbol for the sort* $s$. We write simply $\simeq$ when $s$ is known from the context or irrelevant. A *literal* is either an atom $A$, in which case we call it *positive*, or a negation of an atom $\neg A$, in which case we call it *negative*. When $L$ is a negative literal $\neg A$ and we write $\neg L$, we mean the positive literal $A$. For negative literals with binary predicates $\neg(t_1 \diamondsuit t_2)$ (like, e.g. equality), we sometimes write $t_1 \not\diamondsuit t_2$.

A *clause* is a disjunction of literals $L_1 \vee \ldots \vee L_n$ for $n \geq 0$. We disregard the order of literals and treat a clause as a multiset. When $n = 0$ we speak of the *empty clause*, which is always false. When $n = 1$ a clause is called a *unit clause*. Variables in clauses are considered to be universally quantified. Standard methods exist to transform an arbitrary first-order formula into clausal form (e.g. [15] and our recent work in [19]).

In the following we use *expression* to mean a term, an atom, a literal, or a clause. We write $E[t]_p$ to denote an expression $E$ containing a term $t$ at position $p$ (a position is a unique point in an expression's syntax tree) and may then write $E[s]_p$ to denote the same expression with $t$ replaced by term $s$ at $p$. We will normally leave the position $p$ as implicit. A *substitution* is any $\theta$ of the form $\{x_1 \mapsto t_1, \ldots, x_n \mapsto t_n\}$, where $n \geq 0$. $E\theta$ is the expression obtained from $E$ by the simultaneous replacement of each $x_i$ by $t_i$. An expression is *ground* if it contains no variables. An *instance of E* is any expression $E\theta$ and a *ground instance* of $E$ is any instance of $E$ that is ground. A *unifier* of two terms, atoms or literals $E_1$ and $E_2$ is a substitution $\theta$ such that $E_1\theta = E_2\theta$. It is known that if two expressions have a unifier, then they have a so-called most general unifier.

We assume a standard notion of a (first-order, many-sorted) *interpretation* $\mathcal{I}$, which assigns a non-empty domain $\mathcal{I}_s$ to every sort $s \in \Xi$, and maps every function symbol $f$ to a function $\mathcal{I}_f$ and every predicate symbol $p$ to a relation $\mathcal{I}_p$ on these domains so that the mapping respects sorts. We call $\mathcal{I}_f$ the *interpretation of $f$ in $\mathcal{I}$*, and similarly for $\mathcal{I}_p$ and $\mathcal{I}_s$. Interpretations are also sometimes called *first-order structures.* A *sentence* is a closed formula, i.e. with no free variables. We use the standard notions of validity and satisfiability of sentences in such interpretations. An interpretation is a *model* for a set of clauses if (the universal closure of) each of these clauses is true in the interpretation.

A *theory* $\mathcal{T}$ is identified by a class of interpretations. A sentence is *satisfiable* in $\mathcal{T}$ if it is true in at least one of these interpretations and *valid* if it is true in all of them. A function (or predicate) symbol $f$ is called *uninterpreted* in $\mathcal{T}$, if for every interpretation $\mathcal{I}$ of $\mathcal{T}$ and every interpretation $\mathcal{I}'$ which agrees with $\mathcal{I}$ on all symbols apart from $f$, $\mathcal{I}'$ is also an interpretation of $\mathcal{T}$. A theory is called *complete* if, for every sentence $F$ of this theory, either $F$ or $\neg F$ is valid in this theory. Evidently, every theory of a single interpretation is complete. We can define satisfiability and validity of arbitrary formulas in an interpretation in a standard way by treating free variables as new uninterpreted constants.

The theories we will deal with are the theories of integer, rational, and real arithmetic with uninterpreted functions, denoted by $\mathcal{T}_{\mathbb{Z}}$, $\mathcal{T}_{\mathbb{Q}}$, and $\mathcal{T}_{\mathbb{R}}$, which fix the interpretation of a distinguished sort $\sigma_{\mathbb{Z}}$, $\sigma_{\mathbb{Q}}$, and $\sigma_{\mathbb{R}}$ to the set of mathematical integers $\mathbb{Z}$, rationals $\mathbb{Q}$, and reals $\mathbb{R}$ respectively, and assign the usual meanings to the function and predicate symbols $\{+, -, <, \leq, \cdot\}$. By $\widehat{k}$, we denote the numeral interpreted as $k$ in any of these theories. We consider signatures over these theories to additionally contain uninterpreted functions, and predicates, hence, in contrast to the case without unintpreted functions, for none of these theories there is a sound and complete proof system (see e.g. [13]).

Unless stated differently, we use the symbols $x, y, z$ for variables, $s, t, u$ for terms, $C, D$ for clauses, $p, q, r$ for predicate symbols, $f, g, h$ for function symbols, and $\sigma$ for substitutions, and sorts, with sometimes suffixes being added.

*Term Orderings.* A *simplification ordering* (see, e.g. [8]) on terms is an ordering that is *well-founded*, *monotonic*, *stable under substitutions* and has the *subterm property.* Such an ordering captures a notion of *simplicity*, i.e. $t_1 \prec t_2$ implies that $t_1$ is in some way simpler than $t_2$. Vampire uses the Knuth-Bendix ordering [12], which is parametrized by total precedence ordering on function and predicate symbols $\ll$. This is total on ground terms and partial on non-ground ones, leading to the possibility of *incomparable* terms, e.g. $f(x, a)$ and $f(b, y)$. A simplification ordering $\prec$ on terms can be extended to a simplification ordering on literals and clauses, using a multiset extension of orderings. For simplicity, we will use $\prec$ to refer to the term ordering and its lifting. Whenever $E_1 \prec E_2$ ($E_2 \prec E_1$) we say that $E_1$ is smaller (bigger) than $E_2$. An equality literal $t \simeq s$ is *oriented* if $t \prec s$ or $s \prec t$.

*Saturation-Based Proof Search.* We introduce our new rules within the context of saturation-based proof search. The general idea in saturation is to maintain two sets of *Active* and *Passive* clauses. A saturation-loop then selects a clause $C$ from *Passive*, places $C$ in *Active*, applies *generating inferences* between $C$ and clauses in *Active*, and finally places newly derived clauses in *Passive* after applying some retention tests. The retention tests involve checking whether the new clause is itself redundant (i.e. a tautology) or redundant with respect to existing clauses (implied by a set of smaller clauses in *Active* ∪ *Passive*). Rules that remove the parent clause immediately from the search space without performing a retention test are called immediate simplification rules. Whenever there are applicable immediate simplification rules, the first one wrt. some fixed ordering is chosen to be applied to the selected clause instead of applying any other rule. The rules introduced in this paper are all introduced as immediate simplification rules. However, as mentioned later, not all of them strictly obey the requirement that the result is *smaller*. Normally this would have implications on the *completeness* of the approach but we lose completeness when we start reasoning with theories. This leads us to a trade-off between the potential loss of some proofs by missing some inferences, and the potential gain via simplifying proof search. Our later experimental results show that forgoing completeness is of pragmatic interest.

*Superposition Calculus.* VAMPIRE works with the superposition and resolution calculus (see our previous work [11,14] for a description). The calculus itself is not of direct interest to this work. We do, however, draw attention to two rules. Firstly, the *Equality Resolution* rule

$$\frac{s \not\simeq t \vee C}{C\theta} \quad \theta \text{ is a most general unifier of } s \text{ and } t$$

is a starting point for both our previous theory instantiation work and the Gaussian Variable Elimination rule introduced later (Sec. 3). Secondly, we draw attention to the *Demodulation* (or rewriting by unit equalities) rule

$$\frac{l \simeq r \quad \cancel{L[t] \vee C}}{L[r\theta] \vee C}$$

where $l\theta = t, r\theta \prec l\theta,$ and $(l \simeq r)\theta \prec L[t] \vee C$. This is of interest as later we will need to take special care of the last side-condition when evaluating terms.

*Theory Reasoning.* To perform theory reasoning within this context it is common to do two things. Firstly, to *evaluate* new clauses to put them in a common form (e.g. rewrite all inequalities in terms of $<$) and evaluate ground theory terms and literals (e.g. $1+2$ becomes 3 and $1 < 2$ becomes *false*). More complex evaluation is possible and is the subject of this work (see Section 5). Secondly, relevant theory axioms can be added to the initial search space. For example, if the input clauses use the $+$ symbol one can add the axioms $x + y \simeq y + x$ and $x + 0 \simeq x$, among others.

In addition to these basic methods, VAMPIRE also employs a number of other techniques. *AVATAR modulo theories* [16] uses an SMT solver within the context of clause splitting to ensure that the ground part of any chosen clause splits are theory-consistent. The previously mentioned *unification with abstraction* and *theory instantiation* [20] rules support lazy unification modulo theories and pragmatic instantiation. Theory axiom usage can be controlled by the *set of support*

strategy [17] or layered clause selection [10]. Both approaches de-prioritise reasoning with theory axioms.

## 3 Gaussian variable elimination

Recall the example $7x \neq 6 + y \vee p(y)$ from the Introduction (Sec. 1) where we want to identify the substitution $\{y \mapsto 7x - 6\}$ to produce the simpler instance $p(7x - 6)$. Our general approach is to *rewrite* $7x \neq 6 + y$ in terms of $y$ and then apply the standard *Equality Resolution* rule introduced in Sec. 2. This gives us the straightforward rule:

$$\frac{s \not\simeq t \vee C[x]}{C[u]}\ \mathsf{gve}$$

where $x : \sigma_{\mathbb{Z}}$, $x : \sigma_{\mathbb{Q}}$, or $x : \sigma_{\mathbb{R}}$, $\langle s, t\rangle \Longrightarrow^*_{\mathsf{gve}} \langle x, u\rangle$, or $\langle t, s\rangle \Longrightarrow^*_{\mathsf{gve}} \langle x, u\rangle$ and $x$ is not a subterm of $u$. The relation $\Longrightarrow^*_{\mathsf{gve}}$ is the reflexive, and transitive closure of the relation $\Longrightarrow_{\mathsf{gve}}$ which can be defined as follows.

$$\begin{array}{ll}
\langle s + t, u\rangle \Longrightarrow_{\mathsf{gve}} \langle s, u + (-\, t)\rangle & \\
\langle s + t, u\rangle \Longrightarrow_{\mathsf{gve}} \langle t, u + (-\, s)\rangle & \langle -\, s, t\rangle \Longrightarrow_{\mathsf{gve}} \langle s, -\, t\rangle
\end{array}$$

$$\begin{array}{lll}
\langle s \cdot \widehat{t}, u\rangle & \Longrightarrow_{\mathsf{gve}} \langle s, u \;/\; t\rangle & \text{if } t \neq 0 \text{, and } \widehat{t} : \sigma_{\mathbb{Q}} \text{, or } \widehat{t} : \sigma_{\mathbb{R}} \\
\langle \widehat{s} \cdot t, u\rangle & \Longrightarrow_{\mathsf{gve}} \langle t, u \;/\; s\rangle & \text{if } s \neq 0 \text{, and } \widehat{s} : \sigma_{\mathbb{Q}} \text{, or } \widehat{t} : \sigma_{\mathbb{R}}
\end{array}$$

$$\langle s \;/\; \widehat{t}, u\rangle \Longrightarrow_{\mathsf{gve}} \langle s, u \cdot t\rangle \qquad \text{if } t \neq 0 \text{, and } \widehat{t} : \sigma_{\mathbb{Q}} \text{, or } \widehat{t} : \sigma_{\mathbb{R}}$$

It should be noted that $\Longrightarrow_{\mathsf{gve}}$ is not normalising. The pair $\langle s_1 + s_2, t\rangle$ can, for example, be rewritten to $\langle s_1, t - s_2\rangle$, as well as to $\langle s_2, t - s_1\rangle$. But due to the fact that there is at most a linear number of such rewritings, we can enumerate all of them and choose the first $\langle x, t\rangle$, such that $x$ is not a subterm of $t$. Further choice comes from the fact that we can either rewrite based on $\langle l, r\rangle$, or based on $\langle r, l\rangle$. Looking at our example, we could rewrite

$$\langle 6 + y, 7x\rangle \Longrightarrow_{\mathsf{gve}} \langle y, 7x - 6\rangle$$

but also

$$\langle 7x, 6 + y\rangle \Longrightarrow_{\mathsf{gve}} \langle x, (6 + y) \;/\; 7\rangle$$

if $x$ is not of integer sort, leaving us with a choice. Another source of choice comes from the fact that our premise can contain multiple negative equalities. Any of those could potentially be used to rewrite the rest of the clause.

Since application of the rule, will yield a logically equivalent conclusion, with fewer literals and fewer distinct variables, we make an arbitrary choice. For the same reason, we implement this as a simplification rule (thus removing the premise from the search space) even though the conclusion will often be incomparable to (not smaller than) the premise.

To further demonstrate this rule we consider the additional example

$$\dfrac{\dfrac{\dfrac{\dfrac{p(7xxxy-6)}{p(7xxx-6)}\;\mathsf{asg}^{\cdot}_{\mathsf{var}}}{p(7x-6)}\;\mathsf{asg}^{\mathsf{pow}}_{\mathsf{var}}}{p(x-6)}\;\mathsf{asg}^{\cdot}_{\mathsf{num}}}{p(x)}\;\mathsf{asg}^{+}$$

**Figure 1.** Illustration of the 4 generalization rules, in the theory of Reals.

$$\dfrac{\dfrac{\dfrac{\dfrac{x+y\neq 36 \vee x+3y\neq 90 \vee p(x,y)}{(36-y)+3y\neq 90 \vee p(36-y,y)}\;\mathsf{gve}}{36+2y\neq 90 \vee p(36-y,y)}\;\mathsf{eval}}{\vee\; p(36-(90-36)/2,(90-36)/2)}\;\mathsf{gve}}{p(9,27)}\;\mathsf{eval}$$

which highlights the need to interleave *evaluation* between successive Gaussian elimination steps — we discuss our evaluation strategy below.

## 4 Arithmetic subterm generalization

Taking a closer look at the choice for our example from the previous section, we see that we could have instantiated the premise $y+6 \not\simeq 7x \vee p(y)$ either with $\{y \mapsto 7x-6\}$ to get $p(7x-6)$, or with $\{x \mapsto (6+y)\ /\ 7\}$ to obtain $p(y)$ (again, assuming that $x$ is not of integer sort). Both of the clauses are logically equivalent in $\mathcal{T}_{\mathbb{Q}}$, and $\mathcal{T}_{\mathbb{R}}$, since the earlier is an instance of the latter, and the latter implies the earlier as we can apply the substitution $\{x \mapsto (y+6)\ /\ 7\}$ and simplify the result to the earlier clause. Obviously this kind of reasoning can be applied for any linear subterm $\widehat{k} \cdot x + d$ where $k \neq 0$.

Splitting this idea into multiple rules lets us take these generalizations further. Therefore we propose 4 rules for arithmetic subterm generalization, that are illustrated in a single example in Figure 1.

Since we do not want the applicability of our generalization rules to depend on associativity and commutativity (AC) we will formulate them modulo AC. For this purpose we introduce the following notation. We use $C[t]_{AC}$ to denote a clause that contains the subterm $t$ modulo AC. Further we use $C[t']_{AC}$ to denote the same clause, but all occurrences of $t$ modulo AC, being replaced by $t'$.

*Addition Generalization*

$$\dfrac{C[x+t_1+\ldots+t_n]_{AC}}{C[x]_{AC}}\;\mathsf{asg}^{+}$$

where

- $x : \sigma$ for some $\sigma \in \{\sigma_{\mathbb{Z}}, \sigma_{\mathbb{Q}}, \sigma_{\mathbb{R}}\}$

– all occurrences of $x$ are in the subterm $x + t_1 + \ldots + t_n$ (modulo AC)
– $x$ is not a subterm of $t_i$

The first rule deals with the case where a clause contains a sum with a variable as summand. Such a sum can be generalized by applying the substitution $\{x \mapsto x - t_1 - \ldots - t_n\}$ , and simplifying the result.

*Numeral Multiplication Generalization*

$$\frac{C[\widehat{k} \cdot x \cdot t_1 \cdot \ldots \cdot t_n]_{AC}}{C[x \cdot t_1 \cdot \ldots \cdot t_n]_{AC}} \,\mathsf{asg}^{\cdot}_{\mathsf{num}}$$

where

– $x : \sigma$ for some $\sigma \in \{\sigma_{\mathbb{Q}}, \sigma_{\mathbb{R}}\}$
– all occurrences of $x$ are in the term $\widehat{k} \cdot x \cdot t_1 \cdot \ldots \cdot t_n$ (modulo AC)
– $x$ is not a subterm of $t_i$

In the second rule we generalize a product that contains one variable that occurs only once in this product. Its soundness is justified by the substitution $\{x \mapsto \widehat{\frac{x}{k}}\}$.

*Variable Multiplication Generalization*

$$\frac{C[x \cdot x_1 \cdot \ldots \cdot x_n]_{AC}}{C[x]_{AC}} \,\mathsf{asg}^{\cdot}_{\mathsf{var}}$$

where

– $x : \sigma$ for some $\sigma \in \{\sigma_{\mathbb{Z}}, \sigma_{\mathbb{Q}}, \sigma_{\mathbb{R}}\}$
– all occurrences of $x, x_i$ are in the term $x \cdot x_1 \cdot \ldots \cdot x_n$ (modulo AC)
– $x \neq x_i$

In this rule we generalize subterms that are products of variables, containing redundant variables. The rule is sound since we can replace $x_i$ by $\widehat{1}$.

*Variable Power Generalization*

$$\frac{C[x^n]_{AC}}{C[x^k]_{AC}} \,\mathsf{asg}^{\mathsf{pow}}_{\mathsf{var}}$$

where

– $x : \sigma_{\mathbb{R}}$
– $x^n$ is an abbreviation for $x \cdot x \cdot ... \cdot x$
– $k = \begin{cases} 1 & \text{if } n \text{ is odd} \\ 2 & \text{if } n \text{ is even} \end{cases}$
– all occurrences of $x$ are in the term $x^n$ (modulo AC)

The last rule lets us generalize away redundant powers of variables. Its soundness is guaranteed by the fact, that for Real numbers the co-domains of $x^n$ and $x^k$ are the same.

All of the above rules produce a result that is smaller with respect to any simplification ordering due to the removal of terms, justifying their implementation as immediate simplifications.

## 5 Evaluation

As mentioned above, reasoning with arithmetic often requires us to be able to *evaluate* terms — evaluations such as $3 + 3 \Longrightarrow 6$ and $f(x) + 0 \Longrightarrow f(x)$ are straightforward but we also want to support evaluations such as $(3t+x)+2t \Longrightarrow 5t + x$ for variable $x$ and arbitrary term $t$. We introduce a new method for this (replacing a previous ad-hoc method implemented in VAMPIRE). The general idea is to first rewrite terms into a special *normal form*, apply simplifying steps that preserve this form, and then *denormalise* to obtain standard terms again. We describe the three steps in detail below.

*Normalization.* This step removes the need to take care of reordering and bracketing of terms. Our general normal form is as follows

$$\widehat{c_1} \cdot (t_{1,1} \cdot \ldots \cdot t_{1,k_1}) + \ldots + \widehat{c_n} \cdot (t_{n,1} \cdot \ldots \cdot t_{1,k_n})$$

where $t_{i,j} \prec_1 t_{i,j+1}$ and $(t_{i,1} \cdot \ldots \cdot t_{i,k_i}) \prec_2 (t_{i+1,1} \cdot \ldots \cdot t_{i+1,k_{i+1}})$. To get to this normal form we rewrite $-t$ as $-1 \cdot t$, rewrite $t \;/\; \widehat{c}$ as $t \cdot \widehat{\frac{1}{c}}$, rewrite $t$ as $1 \cdot t$ where necessary, and sort with respect to $\prec_1$ and $\prec_2$. Both relations $\prec_1$, and $\prec_2$ need to be strict total orderings, on terms, and $\prec_1$-sorted lists of terms respectively. VAMPIRE uses so-called aggressive sharing for terms, meaning that for each distinct term there is at most one instance present in memory, and copies are being made by copying the term's id. Hence we can define $\prec_1$ as comparing the ids of two terms. We use the same approach for $\prec_2$.

*Simplification.* Once in normal form, terms can be simplified by joining coefficients for identical terms and removing terms multiplied by zero. This can be given as follows:

$$\widehat{c} \cdot t \cdot \ldots \widehat{d} \ldots \cdot u \Longrightarrow_{\mathsf{eval}} \widehat{cd} \cdot t \cdot \ldots \cdot u$$
$$s + \ldots \widehat{c_1} \cdot t + \widehat{c_2} \cdot t \ldots + u \Longrightarrow_{\mathsf{eval}} s + \ldots \widehat{c_1 + c_2} \cdot t \ldots + u$$
$$s + \ldots + \widehat{0} \cdot t + \ldots + u \Longrightarrow_{\mathsf{eval}} s + \ldots + u$$

If we would generate an empty sum by removing an addition we will simplify to $\widehat{0}$ instead. All of these steps can be implemented in linear time and in a bottom up manner, since we firstly can rely on the terms being sorted by the non-numeral parts of their summands, and secondly on a numeral part of a product being on a fixed position.

*Denormalisation.* Finally, as the normal form contains redundant information (such as $1 \cdot t + \ldots$ instead of $t + \ldots$) we need to *denormalise* as follows:

$$-1 \cdot (t_1 \cdot \ldots \cdot t_n) \Longrightarrow (t_1 \cdot (\ldots \cdot (t_{n-1} \cdot (-\, t_n)) \ldots))$$
$$1 \cdot (t_1 \cdot \ldots \cdot t_n) \Longrightarrow (t_1 \cdot (\ldots \cdot (t_{n-1} \cdot t_n) \ldots))$$

We define the rule $\mathsf{eval}$ to be the chain of normalising, simplifying and denormalising a clause in a bottom-up manner, which is only applied if the step of simplification is successful for some subterm. The reason for not always applying the rules is to prevent arbitrary reordering of sums and products, which in many cases leads to conclusions being bigger than the premise. This can have significant consequences beyond perturbing proof search. Consider the following scenario involving the Demodulation rule (see Sec. 2).

$$\cfrac{x + y \simeq y + x \qquad \cancel{k = a + (b + c)}}{\cfrac{k = a + (c + b)}{k = a + (b + c)}\ \mathsf{eval}}\ \mathsf{demodulation}$$

This process would repeat itself ad infinitum as the initial clause is deleted, replaced by an identical clause. Evaluation would violate the side-condition that should have prevented this, if we would not insist on the step of simplification being successful for the rule to be applied.

In most cases this inference rule is a true simplification wrt. our simplification ordering, since we eliminate at least one symbol in each of the cases in the step *simplification*. Due to generating sometimes bigger terms in the *normalisation*, like in the case $x + x \Rightarrow 1 \cdot x + 1 \cdot x \Rightarrow 2 \cdot x$ we sometimes violate the simplification ordering. Due to the fact that these cases do not occur too frequently, and completeness is not possible in our base theories, we ignore these violations.

During experimentation, we discovered many cases where a unary minus blocks our evaluation rule. Consider the following desired derivation

$$\cfrac{y + t \neq x \vee C[y + -x]}{\cfrac{C[y + -(y + t)}{\cfrac{C[y + (-y + -t)]}{C[t]}}}$$

This is not currently possible as the weight of $-y + -t$ is 5, which is larger than the weight of $-(y + t)$, meaning the second step is not a simplification.

We introduce a simple fix by modifying the weight function and symbol precedence of the Knuth-Bendix ordering as follows:

1. We let $-$ to be weight 0 (for every sorted version of $-$)
2. We let $-$ be the *largest* symbol among symbols of its sort

As a result we can use the following rewrite rule as an additional simplifaction rule, since the right hand side has the same weight as the left hand side, but $-$, the outer most symbol on the left hand side, has higher precedence than $+$ the one on the right hand side.

$$-\ (x + y) \Longrightarrow_{\mathsf{push}^-} (-\ x) + (-\ y)$$

## 6 Cancellation

The motivation for our last rule was two-fold. Firstly evaluation of constant predicates can be helpful in some cases, but fails in seemingly trivial cases. One example for a case like this is the redundant literal $4x + 3 < 4x + 10$. The simple approach of evaluating interpreted predicates fails since we are dealing with non-ground symbols. However it can be simplified to a ground term that can then be evaluated, by cancelling away the $4x$ on both sides of the inequality.

The second motivation were cases where unification with abstraction yields literals in which gve could almost be applied but require a step of cancellation. An example for such a case is the derivation

$$\cfrac{\cfrac{\cfrac{p(5x) \qquad \neg p(3x) \vee C[x]}{3x \neq 5x \vee C[x]}}{0 \neq 2x \vee C[x]}\ \mathsf{cancel}}{C[0]}\ \mathsf{gve}$$

In order to resolve both of these cases we propose the inference rule cancellation cancel, which consists of the following two symmetric cases depending on which side is cancelled.

$$\frac{s + \ldots \widehat{n}t \ldots + u \diamondsuit v + \ldots \widehat{n}t \ldots + w \vee C}{s + \ldots + u \diamondsuit v + \ldots + w \vee C}\ \mathsf{cancel}$$

where

- $\diamondsuit \in \{\simeq, \not\simeq, <, \not<, \leq, \not\leq\}$

$$\frac{s + \ldots \widehat{n}t \ldots + u \diamondsuit v + \ldots \widehat{m}t \ldots + w \vee C}{s + \ldots + u \diamondsuit v + \ldots \widehat{m - n}t \ldots + w \vee C}\ \mathsf{cancel}$$

where

- $\widehat{m - n} \ll \widehat{n - m}$
- $\diamondsuit \in \{\simeq, \not\simeq, <, \not<, \leq, \not\leq\}$

$$\frac{s + \ldots \widehat{n}t \ldots + u \diamondsuit v + \ldots \widehat{m}t \ldots + w \vee C}{s + \ldots \widehat{n - m}t \ldots + u \diamondsuit v + \ldots + w \vee C}\ \mathsf{cancel}$$

where

- $\widehat{n - m} \ll \widehat{m - n}$
- $\diamondsuit \in \{\simeq, \not\simeq, <, \not<, \leq, \not\leq\}$

In order for the rule to not be sensitive to associativity and commutativity, we perform the same steps of normalisation and denormalisation as for the rule eval. Again we will only simplify a clause, if cancellation itself, not only normalisation and denormalisation, is applicable.

The rule is a simplification rule since the number of symbols is reduced with (almost) every application of the cancellation.

**Table 1.** Compares the number of problems solved with any configuration where a new option is enabled to the ones where it is disabled, with a runtime of 10 seconds. The column "both" lists how many were solved in either case. The columns "on", and "off" list how many additional problems could have been solved with the option enabled, or disabled respectively.

| | on | both | off |
|---|---|---|---|
| gve | **121** | 3372 | 104 |
| eval | 323 | 2927 | **347** |
| asg | **440** | 2859 | 298 |
| push$^-$ | **112** | 3378 | 107 |
| cancel | **576** | 2749 | 272 |

## 7 Experimental evaluation

We describe two experiments to establish the impact of the new rules. The first experiment compares the new rules to each other, whilst the second experiment aims to determine how helpful the new rules will be in designing extensions to VAMPIRE's portfolio mode. This is a standard approach to evaluating the benefit of new features in an automated theorem prover [18].

*Experimental Setup.* We implemented the rules as immediate simplification rules in VAMPIRE 4.5 (the implementation is available from the GitHub repository linked from the VAMPIRE website [1], on the branch `integer-arithemtic`). We selected a suitable subset of problems as follows. We started with the set problems of 56,210 from SMT-LIB that involve quantifiers and arithmetic. In a first step we filtered out benchmarks that VAMPIRE could solve within 1 second in **both** default mode (which involves a simpler version of the rule eval), and in default mode with eval enabled. Our main experiments were carried out on the remaining set of 21,512 benchmarks, we which will refer to as **B**. Filtering out trivial benchmarks avoids the results containing noise from benchmarks that can easily be solved and is an approach recently adopted by SMT-COMP [22]. Experiments are run on a Linux cluster where each node contains two octa-core 2.1 GHz Intel Xeon processors and 160GB of RAM. The raw results of our experiments can be found on GitHub[3].

*Experiment 1.* In our first experiment we wanted to find out which are the best combinations of new rules, and whether the rules themselves have a positive impact on proof search. Therefore we ran VAMPIRE in each of the 32 configurations **C** resulting from enabling or disabling each of the 5 groups of rules (asg, gve, eval, push$^-$, and cancel) over **B** with a timeout of 10 seconds.

The results are given in Table 1 showing the total number of problems solved and the problems gained/lost when compared to the default mode with no options set. Each row represents the combination (union) of 16 strategies where

[3] `https://github.com/vprover/vampire_publications/tree/master/experimental_data/TACAS-2021-THEORY-REASONING`

**Table 2.** The top 10 strategies in the greedy ranking of configurations.

| solved | id | eval | gve | asg | push⁻ | cancel |
|---|---|---|---|---|---|---|
| 2546 | 15 | ✓ | ✓ | ✓ | ✓ | ✓ |
| 548 | 24 | | ✓ | | | |
| 136 | 27 | | ✓ | | ✓ | ✓ |
| 63 | 22 | | | ✓ | ✓ | |
| 51 | 9 | ✓ | ✓ | | | ✓ |
| 38 | 4 | ✓ | | ✓ | | |
| 27 | 23 | | | ✓ | ✓ | ✓ |
| 20 | 26 | | ✓ | | ✓ | |
| 19 | 25 | | ✓ | | | ✓ |
| 18 | 5 | ✓ | | ✓ | | ✓ |

**Table 3.** The symmetric difference in number of problems solved between the three new strategies in portfolio mode against Vampire 4.5. Each cell indicates the number of problems solved by the row solver unsolved by the column solver. The column unique lists how many problems each strategy could solve that no other strategy could. The strategy Vampire * is what we can solve with either of the three other strategies. Vampire * is not taken into account for uniqueness.

| strategy | total | unique | Vampire * | 15 | 24 | 27 | Vampire 4.5 |
|---|---|---|---|---|---|---|---|
| Vampire * | 7511 | 0 | 0 | 622 | 937 | 932 | 1052 |
| 15 | 6889 | 64 | 0 | 0 | 865 | 729 | 824 |
| 24 | 6574 | 12 | 0 | 550 | 0 | 261 | 366 |
| 27 | 6579 | 2 | 0 | 419 | 266 | 0 | 165 |
| Vampire 4.5 | 6506 | 10 | 47 | 441 | 298 | 92 | 0 |

that option is turned on. This shows that, with the exception of evaluation, the gains outweigh the losses, sometimes considerably. This result for evaluation tells us that the other rules can still operate effectively without our new evaluation and, further, that the two evaluation methods are in some sense complementary. Therefore, whilst we explore this further, we will keep both evaluation methods. The most significant gains are with cancellation, which may be related to the fact that it is applicable to inequalities as well as equalities.

*Greedy Ranking.* Another way of looking at the results of Experiment 1 is to create a greedy ranking *rank* of all configurations **C**, starting with the set of all configurations, and ranking the configuration solving the most benchmarks in **B** as the best, ranking the one that solves most of the remaining benchmarks as second, and so on. The top 10 strategies in this ranking are given in Table 2. The overall best strategy uses all 5 of the new rules. Interestingly, the second best strategy only uses the gve rule. This ranking indicates the most promising strategies to use in our next experiment.

*Experiment 2* In our second experiment we wanted to see how many new problems we can solve with the new simplification rules compared to our current

**Table 4.** Comparing our new approach, Vampire *, against Vampire 4.5, Cvc4, and Z3 with results separated by logic. The notation $(+a, -b)$ means that the solver solved $a$ problems the new Vampire could not solve, and the new vampire could solve $b$ the other solver couldn't. The entries $a(b)$ in the column Vampire *, list the number $a$ of problems that could be solved by our new rules, and $b$ the number of these problems that could not be solved by any of the other solvers.

| | count | Vampire * | Vampire 4.5 | Cvc4 | Z3 |
|---|---|---|---|---|---|
| ALIA | 24 | 14 (0) | 12 (+0, -2) | 23 (+9, -0) | **24** (+10, -0) |
| AUFDTLIA | 134 | 39 (0) | 39 (+0, -0) | **86** (+47, -0) | 80 (+45, -4) |
| AUFLIA | 862 | 312 (4) | 311 (+4, -5) | 295 (+84, -101) | **331** (+148, -129) |
| AUFLIRA | 1697 | 1364 (0) | 1354 (+0, -10) | **1455** (+101, -10) | 1453 (+102, -13) |
| AUFNIA | 3 | **0** (0) | **0** (+0, -0) | **0** (+0, -0) | **0** (+0, -0) |
| AUFNIRA | 509 | **87** (2) | 81 (+2, -8) | **87** (+20, -20) | 63 (+16, -40) |
| LIA | 246 | 79 (0) | 78 (+0, -1) | **246** (+167, -0) | 230 (+155, -4) |
| LRA | 2043 | 1013 (41) | 365 (+0, -648) | 1528 (+635, -120) | **1756** (+883, -140) |
| NIA | 11 | 1 (0) | 1 (+0, -0) | **9** (+9, -1) | 5 (+4, -0) |
| NRA | 101 | 92 (0) | 91 (+0, -1) | 72 (+0, -20) | **96** (+9, -5) |
| UFDTLIA | 274 | **120** (4) | 115 (+0, -5) | 40 (+3, -83) | 34 (+1, -87) |
| UFDTLIRA | 33 | 0 (0) | 0 (+0, -0) | **33** (+33, -0) | **33** (+33, -0) |
| UFLIA | 4833 | 1924 (23) | 1829 (+30, -125) | **2314** (+501, -111) | 1899 (+315, -340) |
| UFLRA | 7 | 2 (0) | 2 (+0, -0) | 2 (+0, -0) | **5** (+3, -0) |
| UFNIA | 10735 | 2463 (16) | 2227 (+11, -247) | **4928** (+3055, -590) | 3858 (+1983, -588) |
| Any Logic | 21512 | 7510 (90) | 6505 (+47, -1052) | **11118** (+4664, -1056) | 9867 (+3707, -1350) |

best effort in Vampire 4.5. Therefore we ran Vampire with the three top ranking configurations of experiment 3 forced added on top of Vampire's portfolio mode. The portfolio mode executes a sequence of strategies heuristically chosen based on problem features. Forcing a configuration of new options on top of this forces each strategy to make use of the new options. We ran this experiment over **B** with a timeout of 200 seconds.

Results are given in Table 3 and show that the new rules allow Vampire to solver considerably more problems (1052) than it could before whilst losing relatively few (47). The best configuration of options (all five new rules) solves the most with the other two configurations solving roughly the same. The interesting point here is that they remain complementary, solving a large number of problems uniquely. These are the exact conditions we require for producing a new, powerful portfolio mode. It is likely that performance will improve even further when also considering other option combinations.

Finally, Table 4 compares the number of problems solved by either of the three top strategies – referred to as Vampire∗ – against Vampire 4.5, Z3 [7] and Cvc4 [4]. Results are further separated by the logic in which the benchmarks belong — A stands for Arrays, UF stands for Uninterpreted Functions, DT stands for Data Types, L stands for Linear, N for Non-linear, I stands for Integers, R stands for Reals, with the final A standing for Arithmetic. Here we notice that the new rules make a considerable impact in the case of pure linear real arithmetic. This is likely due to the fact that the asg allows us to fully generalise away most linear terms and gve will be broadly applicable without uninterpreted functions. It is interesting to note that, whilst the new Vampire solves fewer problems than Cvc4, and Z3 overall, it solves many (1056, and 1350) problems that the other provers do not solve. The most striking result is that we can solve 90 new problems, neither Vampire 4.5 nor either of the state-of-the-art SMT solvers could solve.

## 8 Conclusion

We have motivated and introduced five new simplification rules for reasoning in the theory of arithmetic within saturation-based first-order theorem provers. These rules were implemented within the Vampire theorem prover and demonstrated to improve the reasoning power on problems taken from SMT-LIB. It remains future work to explore the ideal combinations of these rules and existing proof search heuristics. It also remains an open question whether we can design an evaluation rule and modified simplification ordering that ensures that every evaluation that we want to perform is a true simplification. As demonstrated, this is not necessary pragmatically but would be satisfying theoretically.
